# Long-term Metformin Alters Gut Microbiota and Serum Metabolome in Coronary Artery Disease Patients After Percutaneous Coronary Intervention to Improve 5-year Prognoses: A Multi-omics Analysis

**DOI:** 10.31083/RCM26835

**Published:** 2025-05-27

**Authors:** Ruilin Zhou, Qingyang Wu, Hao Qian, Liang Wang, Guangcheng Liu, Bin Zhang, Wei Wu, Shuyang Zhang

**Affiliations:** ^1^Department of Cardiology Medicine, Peking Union Medical College Hospital, Chinese Academy of Medical Science and Peking Union Medical College, 100730 Beijing, China; ^2^Eight-Year Medical Doctor Program, Chinese Academy of Medical Sciences and Peking Union Medical College, 100730 Beijing, China; ^3^Department of Cardiology, State Key Laboratory of Complex Severe and Rare Diseases, Peking Union Medical College Hospital, Chinese Academy of Medical Sciences & Peking Union Medical College, 100730 Beijing, China

**Keywords:** metformin, gut microbiota, coronary artery disease, diabetes mellitus, multiomic analyses

## Abstract

**Background:**

About 20% of patients with coronary artery disease (CAD) experience adverse events within five years of undergoing percutaneous coronary intervention (PCI) for acute myocardial infarction. In these patients, the impact of metformin on long-term prognosis remains uncertain.

**Methods:**

This study enrolled 22 metformin (Met)-CAD patients with diabetes mellitus (DM) who had been administered metformin for at least six months before PCI, 14 non-Met CAD-DM patients with DM who had never taken metformin or had stopped taking metformin for a year before PCI, and 22 matched healthy controls. A 5-year follow-up was conducted to collect clinical prognosis data. Fecal 16S rRNA sequencing and serum untargeted metabolomics analyses were performed. BugBase was utilized to analyze the possible functional changes in the gut microbiome. Multi-omics analysis was conducted using Spearman’s correlation to explore the interactions between metformin, gut microbiome, serum metabolites, and clinical prognosis.

**Results:**

Metformin significantly lowered the 5-year major adverse cardiac events (MACEs) in Met CAD-DM patients. We found a higher abundance of *Bacteroides coprocola*, *Bacteroides massiliensis*, *Phascolarctobacterium succinatutens*, and *Eubacterium coprostanoligenes* in the Met CAD-DM patients, as well as an increase in hydroxy-alpha-sanshool (HAS) and decenoylcarnitine and a decrease in tridec-10-enoic acid, Z-vad-fmk (benzyloxycarbonyl–Val–Ala–Asp (OMe)–fluoromethylketone), 3,9-dimethyluric acid in blood serum. Multi-omics analysis revealed that alterations in the gut microbiome and serum metabolites are significantly associated with the 5-year prognosis of CAD-DM.

**Conclusions:**

Metformin significantly improved the 5-year prognosis of CAD patients following PCI. Metformin tended to have more positive effects on the commensal flora and metabolic profiles, which may explain its beneficial effects on cardiovascular health. This study revealed the potential associations between metformin and the gut microbiome, an associated alteration in serum metabolome, and the impact on the host immune system and metabolic pathways.

## 1. Introduction

Coronary artery disease (CAD) is a major global health concern and a leading cause of mortality worldwide [1]. Among CAD patients who underwent percutaneous coronary intervention (PCI), approximately 20% experienced adverse events within five years, with an all-cause mortality rate ranging from 2% to 11% following stent implantation [2]. Chronic inflammation is a key driver of atherosclerosis progression and plaque destabilization in diabetes mellitus (DM). This inflammatory milieu promotes adverse remodeling of coronary arteries, thereby increasing the risk of adverse events in CAD patients. Chronic low-grade inflammation plays a pivotal role in the pathogenesis of coronary artery disease [3]. The activation of the inflammatory response significantly contributes to plaque destabilization and subsequent development of acute coronary syndromes [4]. Several signaling pathways associated with the inflammatory response, including the nucleotide-binding oligomerization domain (NOD)-, leucine-rich repeat (LRR)- and pyrin domain-containing protein 3 (NLRP3) inflammasome, toll-like receptors, and Notch and Wnt signaling pathways, have been implicated in the development and subsequent regression of atherosclerosis [5]. The polarization of M1 macrophages is considered to be involved in this process. M1 macrophages are primarily activated by lipopolysaccharide (LPS) and γ-interferon (IFNγ), which engage Toll-like receptor 4 (TLR4) and γ-interferon receptor (IFNγR). This activation leads to the up-regulation of key signaling pathways, including nuclear factor-κB (NF-κB), Janus kinase (JAK)-signaling, and the signal transducer and activator of transcription 1 (STAT1), which collectively enhance the secretion of pro-inflammatory factors. These mediators contribute to the promotion of inflammation, pathogen clearance, and phagocytosis [5]. Therapeutic interventions focusing on anti-inflammation have shown promising results. For example, a phase I randomized trial of dapansutrile (a specific NLRP3 inflammasome inhibitor) demonstrated its safety in heart failure patients with reduced left ventricular ejection fraction [6]. Chronic inflammation plays an important role in the development of coronary artery disease and diabetes mellitus, and finding new therapeutic targets to down-regulate inflammation appears to be a promising approach.

In recent years, the gut microbiota has received significant attention due to its potential involvement in various disease mechanisms [7, 8]. There is growing evidence to suggest that changes in the structure and function of the gut microbiota contribute to the onset and progression of CAD [9] and DM [10]. The gut microbiota can break down dietary components and generate metabolites that affect the host’s metabolism and immune responses [11]. These metabolites can influence various physiological functions and contribute to disease progression. An imbalance in the gut microbiota can increase inflammation, triggering immune activation and chronic inflammation [12]. This prolonged inflammatory state plays a crucial role in the development of numerous diseases [13]. In addition, disruptions in the gut microbiota can compromise the integrity of the gut barrier, leading to increased permeability and translocation of microbial byproducts. This process activates immune responses and may contribute to the emergence of systemic diseases. Therefore, exploring the connection between the gut microbiota and the progression of CAD and DM offers a novel perspective on understanding these diseases.

Metformin is a widely prescribed oral antidiabetic medication. In recent years, metformin was found to be beneficial for cardiovascular health. Metformin has been found to have beneficial effects on body mass index (BMI) and blood pressure, thereby helping to promote cardiovascular health [14]. Metformin also plays a crucial role in modulating the gut microbiota [15]. Metformin also affects microbial metabolism and virulence factors, which are essential for maintaining gut homeostasis. It has been suggested that metformin could influence the gut microbiota through various mechanisms. Metformin results in alterations to the structure of the gut microbiota by metabolic changes which may enhance the presence of beneficial bacteria, such as lactobacilli, and decreasing harmful bacteria [16, 17, 18]. Alternatively, metformin can affect microbial metabolism, stimulating the generation of metabolites such as short-chain fatty acids (SCFAs) which are crucial for maintenance of gut health [19]. Furthermore, a study showed that metformin may play a role in reducing the virulence of specific pathogenic bacteria, thereby decreasing inflammation [20]. However, research has primarily focused on the short-term effects of metformin on the gut microbiome. Metformin has been widely used as a first-line drug in diabetes and is often used long-term. Long-term use of metformin has a significant effect on the health of the host and their gut microbiome. However, the long-term effects of metformin have not been thoroughly investigated. Therefore, examining the mechanisms underlying CAD complicated by DM, along with alterations in gut microbiota linked to long-term metformin use, is of immense value.

## 2. Methods

### 2.1 Study Population

The participants in the study were consecutively recruited at the Department of Cardiology in the Peking Union Medical College Hospital from 2016 to 2018. The inclusion criteria required patients to have ≥50% stenosis in at least one main coronary artery as identified by coronary angiography. Exclusion criteria included a history of gastrointestinal diseases, malignant tumors, autoimmune disorders, infectious diseases, and renal dysfunction (creatine >3 mg/dL). Patients were also excluded if they had undergone gastrointestinal surgery within the past year, or had received antibiotics for more than three days within the previous three months. Peripheral venous blood and stool samples were collected the morning after admission and processed as previously described [21]. The freshly collected samples from each participant were immediately transported to our laboratory and stored at –80 ℃. During the storage and transportation process, the fecal samples were kept and transported in dry ice at –78.5 ℃.

Data on metformin intake was collected. Typically, the gut microbiota undergoes significant changes within three months following the onset of the disease or alternations in medication, and these changes are maintained thereafter [22, 23]. Therefore, based on metformin usage for at least six months, the 36 CAD patients with DM were divided into two groups: (1) Metformin (Met) CAD-DM group (N = 22): CAD-DM patients who had taken metformin for at least six months before PCI. (2) Non-Met CAD-DM group (N = 14): CAD-DM patients who had never taken metformin or had stopped taking metformin for at least one year before PCI.

In addition, 22 healthy volunteers who met the following criteria were enrolled as the healthy control (HC): (1) did not take metformin, (2) did not suffer from CAD or DM, and (3) did not meet any of the above exclusion criteria.

All patients underwent 5-year follow-up. All the enrolled patients developed no additional comorbidities or changes in medication throughout the 5-year follow up period.

Written informed consent was obtained from all participants and the study adhered to the principles of the Declaration of Helsinki. The ethical approval was obtained from the Peking Union Medical College Hospital, and the protocol number is I-24PJ0927.

### 2.2 16S rRNA Gene V3–V4 Region Sequencing of Gut Microbiota

The samples were prepared and stored according to the protocol described in our previous study [21]. Microbial DNA was extracted from the fecal samples using the bead-beating method [24]. PCR was conducted to amplify the V3–V4 region of 16S rRNA genes [25]. The sequencing library was established as described previously [26], and purified products were sequenced using the Illumina Miseq system (Illumina Inc., San Diego, CA, USA). The downstream analysis of amplicons was performed using EasyAmplicon v1.0 (Fred Hutchinson Cancer Research Center, Seattle, WA, USA) [27]. For dereplication, the derep_fullength command in VSEARCH v2.15 (Technical University of Denmark (DTU), University of Copenhagen, Copenhagen University Hospital, Copenhagen, Denmark) was employed [28]. Operational taxonomic units (OTUs) were grouped using the -cluster_otus command in USEARCH (v10.0, Illumina, Inc, San Diego, CA, USA) with a 97% cutoff [29]. A feature table was generated using vsearch–usearch_global, and taxonomic classification was performed based on the Greengenes database using usearch–otutab [30].

All sample sequences were normalized to match the sample with the fewest (10,560) sequences for diversity index calculations. An observed species richness index was used to assess alpha diversity. Beta diversity was examined through principal coordinate analysis (PCoA) and constrained PCoA (CPCoA) using Bray-Curtis distances. Group compositions were visualized at the phylum level as boxplots and at the genus level as a Chord diagram using the R package ggplot2 (https://ggplot2.tidyverse.org/). To compare differences, edgeR (https://bioconductor.org/packages/release/bioc/html/edgeR.html) was utilized to detect group variances with the application of negative binomial distribution, and the Benjamini-Hochberg method controlled the false discovery rate (FDR) [31]. A significance level of *p* < 0.05 with a FDR <0.2 is considered as statistically significant. Bugbase was employed for functional prediction of gut microbiota [32]. Differences in pathways were pinpointed using Welch’s *t*-test after normalization, and Storey FDR was applied for multiple pathways. The STAMP software (v2.1.3, National Institute of Genetics, Mishima, Japan) facilitated statistical analysis and visualization of these pathways.

### 2.3 Identification of the Key Microbes or Metabolites Associated With Metformin Intake

A Wilcoxon rank-sum test was used for metabolomics analysis. Differential metabolites were identified based on a Variable Importance in the Projection (VIP) greater than 1 and a significance level of *p* < 0.05. Metabolites showing significantly higher or lower levels in the Met CAD-DM group compared to both the non-Met CAD-DM and HC groups were considered key metabolites associated with metformin intake. Using edgeR with a threshold of *p* < 0.05 revealed 32 distinct OTUs between Met CAD-DM and non-Met CAD-DM. Key OTUs exhibited the highest or lowest average relative abundance in Met CAD-DM compared to non-Met CAD-DM and HC.

### 2.4 Untargeted Metabolomics Analysis

Serum metabolome analysis was performed using a Waters ACQUITY ultra-high-performance liquid chromatography system (Milford, MA, USA) in conjunction with a Waters Q-TOF Micromass system (Manchester, UK) operating in both positive and negative ionization modes. Different modes, such as polar ionic and lipid modes, were used based on the metabolite characteristics. The sample preparation and experimental procedures for liquid chromatograph-mass spectrometer (LC-MS) were previously outlined and explained. A peak-ion intensity matrix was refined by eliminating peaks that displayed zero values in more than 80% of samples. A quality control sample coefficient of variation threshold of 30% was implemented. To identify metabolites showing significant differences between groups, a Wilcoxon rank-sum test was used. Subsequently, partial least squares discriminant analysis (PLS-DA) was conducted via SIMCA software (MKS Umetrics, Uppsala, Sweden). Peaks were considered important based on a VIP-value greater than 1 and a significance level of *p* < 0.05. All *p*-values were FDR-adjusted. Online databases such as the Human Metabolome Database, LipidMaps, and PubChem were used to categorize peaks according to their molecular mass data (m/z). Pathway enrichment analysis was performed using MetaboAnalyst (University of Alberta​ in ​Edmonton, Alberta, Canada; https://www.metaboanalyst.ca/), identifying pathways with an impact-value exceeding 0.10 as potential targets [33].

### 2.5 Statistical Analysis, Multi-Omics Correlation Study and Visualization

One-way ANOVA was used for analyzing continuous data that followed a normal distribution across the three groups. Non-normally distributed continuous data among three groups were analyzed using the Kruskal-Wallis H-test, while comparisons between two groups were conducted using the Mann-Whitney U test. Categorical variables were assessed through either the χ^2^ test or Fisher’s exact test. The data analysis was carried out using SPSS (v.24.0, IBM Corp., Armonk, NY, USA), and figures were visualized using heatmaps generated with the R package heatmap. Spearman correlation analysis was conducted using SPSS (v.24.0) to examine the relationships among key bacterial taxa, serum metabolites, and clinical parameters. The results were visualized as a heatmap using the R package heatmap.

## 3. Results

### 3.1 Metformin Significantly Improves 5-year Prognosis in Patients With Coronary Artery Disease With Diabetes Mellitus

The 36 CAD patients with diabetes were divided into two groups: those who took metformin (Met CAD-DM group, N = 22) and those who did not take metformin (non-Met CAD-DM group, N = 14). Both stool and blood samples were collected in 2019. At the time of sample collection, patients in the Met CAD-DM group had been taking metformin for at least one year and continued taking it for the following five years. In contrast, patients in the non-Met CAD-DM group did not take metformin for one year before fecal collection and did not take metformin for the subsequent five years. The clinical characteristics of the study cohort are presented in Table 1. Baseline clinical characteristics, including age, BMI, blood pressure, comorbidities (HTN (hypertension), HLP (hyperlipidemic pancreatitis), FLD (fatty liver disease)), CAD severity (Gensini score, New York Heart Association (NYHA) score), and cardiac biomarkers (CK-MB (creatine kinase MB isoenzyme), hsCRP (high sensitivity C-reactive protein)), were comparable between the two groups.

**Table 1.** **Comparison of clinical features and laboratory data between three groups**.

		HC	Met CAD-DM	Non-Met CAD-DM	*p*-value
		(N = 22)	(N = 22)	(N = 14)	
Demographics					
	Age^*^	55.86 ± 7.77	64.82 ± 8.86	64.29 ± 11.01	0.003^a⁢b^
	Gender (Male)^§^	11 (50.0)	15 (68.2)	9 (64.3)	0.441
	SBP, mmHg^*^	117.86 ± 9.10	131.36 ± 14.81	127.43 ± 16.37	0.003^a^
	DBP, mmHg^†^	76.00 (20.50)	73.00 (16.75)	75.00 (13.25)	0.912
	Height, cm^*^	165.27 ± 9.51	164.73 ± 8.30	166.29 ± 7.61	0.870
	Weight, kg^*^	66.03 ± 9.74	75.16 ± 9.64	72.71 ± 14.19	0.014^a^
	BMI, kg/m^2⁣*^	24.14 ± 2.67	27.39 ± 2.99	25.97 ± 3.58	0.003^a^
	Waist, cm^*^	82.14 ± 8.58	95.64 ± 7.15	93.29 ± 9.77	<0.001^a⁢b^
	Gensini Score^*^	NA	41.48 (24.59)	43.85 (37.85)	0.823
	Smoke^§^	4 (18.2)	12 (54.5)	5 (35.7)	0.043^b^
	Drink^§^	1 (4.5)	13 (59.1)	4 (28.6)	<0.001^a⁢b^
	Family history^§^	7 (31.8)	10 (45.5)	6 (42.9)	0.627
NYHA					0.397
	I^§^	NA	10 (45.5)	9 (64.3)	
	II^§^	NA	11 (50.0)	4 (28.6)	
	III^§^	NA	1 (4.5)	1 (7.1)	
	IV^§^	NA	0 (0.0)	0 (0.0)	
Past/Personal history					
	OMI^§^	0 (0.0)	1 (4.5)	2 (14.3)	0.241
	PAS^§^	2 (9.1)	6 (27.3)	4 (28.6)	0.250
	TGD^§^	2 (9.1)	3 (13.6)	3 (21.4)	0.562
	FLD^§^	6 (27.3)	7 (31.8)	4 (28.6)	0.945
	HLP^§^	7 (31.8)	19 (86.4)	10 (71.4)	<0.001^a⁢b^
	HTN^§^	6 (27.3)	15 (68.2)	9 (64.3)	0.014^a⁢b^
Laboratory data					
	cTnI^†^	NA	0.01 (0.05)	0.01 (0.26)	0.510
	CK, U/L^†^	111.00 (40.75)	91.50 (48.00)	108.50 (60.75)	0.046^a^
	CK-MB, U/L^†^	0.90 (0.63)	0.70 (0.53)	0.70 (0.70)	0.810
	TC, mmol/L^*^	4.79 ± 0.86	4.37 ± 1.39	4.04 ± 1.57	0.218
	TG, mmol/L^†^	1.37 (1.36)	1.63 (1.49)	1.16 (1.33)	0.389
	LDL-C, mmol/L^†^	2.76 (0.81)	2.19 (1.29)	1.73 (1.23)	0.034^b^
	HDL-C, mmol/L^†^	1.20 (0.40)	0.91 (0.23)	0.97 (0.26)	0.002^a⁢b^
	hsCRP, mg/L^†^	0.70 (0.77)	2.78 (3.03)	2.09 (2.41)	<0.001^a⁢b^
	ALT, U/L^†^	18.00 (13.25)	25.00 (16.25)	22.00 (21.25)	0.140
	AST, U/L^†^	NA	23.00 (5.50)	23.00 (23.00)	0.693
	GGT, U/L^†^	NA	32.00 (19.25)	22.00 (16.50)	0.151
	ALP, U/L^*^	NA	75.36 ± 14.76	72.62 ± 11.00	0.565
	LDH, U/L^†^	NA	162.50 (29.50)	193.00 (50.50)	0.006
	TBil, µmol/L^*^	14.68 ± 4.03	11.35 ± 4.89	10.36 ± 4.08	0.009^a⁢b^
	DBil, µmol/L^*^	4.79 ± 1.79	3.22 ± 1.37	2.94 ± 1.08	<0.001^a⁢b^
	Cr, µmol/L^†^	71.00 (20.50)	69.50 (24.25)	76.50 (23.25)	0.735
	Urea, mmol/L^*^	4.82 ± 0.93	6.49 ± 1.41	5.44 ± 2.02	0.001^a^
	HGB, g/L^*^	145.23 ± 15.06	136.41 ± 16.43	136.36 ± 8.27	0.084
	WBC, ×10^9^/L^*^	5.37 ± 1.36	7.20 ± 1.67	5.83 ± 1.63	<0.001^a⁢c^
	RBC, ×10^9^/L^†^	4.60 (0.47)	4.46 (0.39)	4.47 (0.49)	0.248
	HCT, %^*^	41.97 ± 3.60	39.71 ± 4.57	40.00 ± 2.74	0.124
	Glucose, mmol/L^†^	5.90 (1.95)	9.20 (5.38)	8.10 (3.43)	<0.001^a⁢b^
	IL-18, U/L (pg/mL)^*^	861.16 ± 277.60	678.34 ± 515.98	717.90 ± 341.20	0.236
	IL-1β^†^	3.26 (0.65)	3.00 (1.15)	3.04 (3.22)	0.886
	IL-6^†^	2.81 (1.88)	4.15 (9.64)	3.48 (10.61)	0.044^b^
	TNF-α^†^	3.87 (9.77)	19.88 (30.39)	27.61 (41.55)	<0.001^a⁢b^

^*^, mean ± SD; ^§^, n (%); ^†^, median (IQR); NA, not available. ^a^*p* < 0.05 stands for significant difference between HC vs. Met CAD-DM. ^b^*p* < 0.05 stands for significant difference between HC vs. Non-Met CAD-DM. ^c^*p* < 0.05 stands for significant difference Met CAD-DM vs. Non-Met CAD-DM. HC, healthy control; Met, metformin; CAD, cardiovascular disease; DM, diabetes mellitus; SBP, systolic blood pressure; BMI, body mass index; OMI, old myocardial infarction; PAS, peripheral atherosclerosis; TGD, thyroid gland dysfunction; FLD, fatty liver disease; HLP, hyperlipidemia; HTN, hypertension; cTnI, cardiac troponin I; CK, creatine kinase; CK-MB, creatine kinase MB isoenzyme; TC, total cholesterol; TG, total triglyceride; LDL-C, low density lipoprotein-cholesterol; HDL-C, high density lipoprotein-cholesterol; hsCRP, high sensitivity C-reactive protein; ALT, alanine transaminase; AST, aspartate transaminase; GGT, gamma-glutamyl transferase; ALP, alkaline phosphatase; LDH, lactate dehydrogenase; TBil, total bilirubin; DBil, direct bilirubin; Cr, creatine; HGB, hemoglobulin; WBC, white blood cells; RBC, red blood cells; HCT, hematocrit value; IL, interleukin; TNF, tumor necrosis factor; DBP, diastolic blood pressure; IQR, interquartile range; NYHA, New York Heart Association.

After five years, in March 2024, we conducted a follow-up assessment with all the patients via telephone. The results showed that of the 22 individuals in the Met CAD-DM group, six patients had a major adverse cardiac event (MACE) event within five years, whereas out of the 14 individuals in non-Met CAD-DM group, nine had a MACE event. These results were statistically significant (*p* = 0.03). This indicates that patients in the Met CAD-DM group have a significantly better clinical prognosis than those in the non-Met CAD-DM group, indicating that metformin significantly enhanced the 5-year cardiovascular prognosis.

### 3.2 Metformin Significantly Alters the Taxonomic Features of Gut Microbiome

**Fig. 1.**
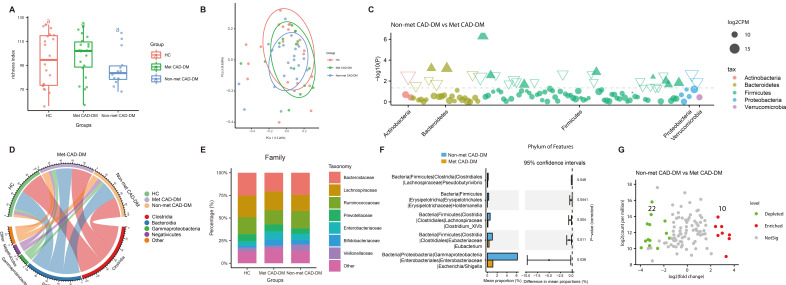
**Medication of metformin affects the taxonomic features of gut microbiota in patients with CAD-DM**. (A) Observed Species Richness Index, representing the number of operational taxonomic units (OTUs) identified in each group. Box-plot features represent the mean ± SD level. (B) Beta diversity analyzed by constrained PCoAs (CPCoA) plot based on Bray-Curtis distances. (PCo1 15.26%, PCo2 9.926%). (C) Manhattan plot demonstrating the differentially abundant gut microbes and their contributions to each phylum. Filled triangles, hollow inverted triangles, and solid circles indicate OTUs enriched, depleted, and without significant difference, respectively. The color of each marker represents the different taxonomic affiliation of the OTUs, and the size corresponds to their relative abundances using log2 transformed counts per million (CPM) values. (D) Chord plot showing the dominant classes and their contribution to each group. (E) Relative abundances of bacteria among groups at the family level. (F) Differential gut microbes between the non-Met CAD-DM group and the met CAD-DM group at the genus level. (G) Volcano plots showing differential gut microbes at the species level in patients between the non-Met CAD-DM group and the met CAD-DM group. “a” means there isn’t a significant difference. PCol, principal co-ordinates 1; PCoA, principal coordinate analysis.

Despite similar baseline clinical characteristics, the Met CAD-DM group exhibited a significantly improved five-year clinical prognosis compared to the non-Met CAD-DM group. To investigate the potential role of the gut microbiome on this outcome, 16S rRNA sequencing was performed (**Supplementary Table 1 **and **Supplementary Table 2)**. Alpha diversity analysis revealed a higher gut microbiome diversity in the healthy control group compared to both patient groups, with the lowest diversity observed in the non-Met CAD-DM group (Fig. 1A). Beta diversity analysis demonstrated microbial community structures across the three groups (Fig. 1B, **Supplementary Fig. 1**).

We further analyzed the specific structural composition of the three groups. The results showed that in the phylum level of gut microbiota (Fig. 1C), the non-Met CAD-DM group exhibited a greater incidence of *Firmicutes *compared to the Met CAD-DM group. At the class level of gut microbiota (Fig. 1D), *Clostridia* constituted the largest proportion across all three groups, followed by *Bacteroidia*. The proportion of *Clostridia* in the Met CAD-DM group and non-Met CAD-DM group were very similar but was much lower compared to the HC. The three least abundant classes were *Gammaproteobacteria*, *Negativicutes*, and Others. These three classes also had different proportions in the non-Met CAD-DM group, Met CAD-DM group, and HC group. At the family level of gut microbiota (Fig. 1E), *Bacteroidaceae* showed the highest abundance in all three groups, followed by *Lachnospiraceae*, *Ruminococcaceae*, *Prevotellaceae*, *Enterobacteriaceae*, *Bifidobacteriaceae*, and *Veillonellaceae*. We then compared the genus level of gut microbiota between the non-Met CAD-DM group and the Met CAD-DM group (Fig. 1F). The differential genera included *Escherichia/Shigella*, *Eubacterium*, *Clostridium_XlVb*, *Holdemanella*, and *Pseudobutyrivibrio*. To further investigate the role of metformin in shaping gut microbiome, we analyzed the differential species between the non-Met CAD-DM group and the Met CAD-DM group (Fig. 1G). The analysis identified a total of 32 distinct gut microbiome species between the two disease groups. The specific FDR-adjusted *p*-values are presented in **Supplementary Table 3**. 10 species were more enriched in the Met CAD-DM group than in the non-Met CAD-DM group, while 22 species were more enriched in the non-Met CAD-DM group than in the Met CAD-DM group. These results demonstrate that metformin significantly alters the abundance and composition of gut microbiota. The clinical data indicate no baseline differences between groups, suggesting that the observed microbial changes are likely due to metformin treatment rather than pre-existing variability.

### 3.3 The Reshape of Gut Microbiome by Metformin is Closely Associated With Patients’ Clinical Prognosis

**Fig. 2.**
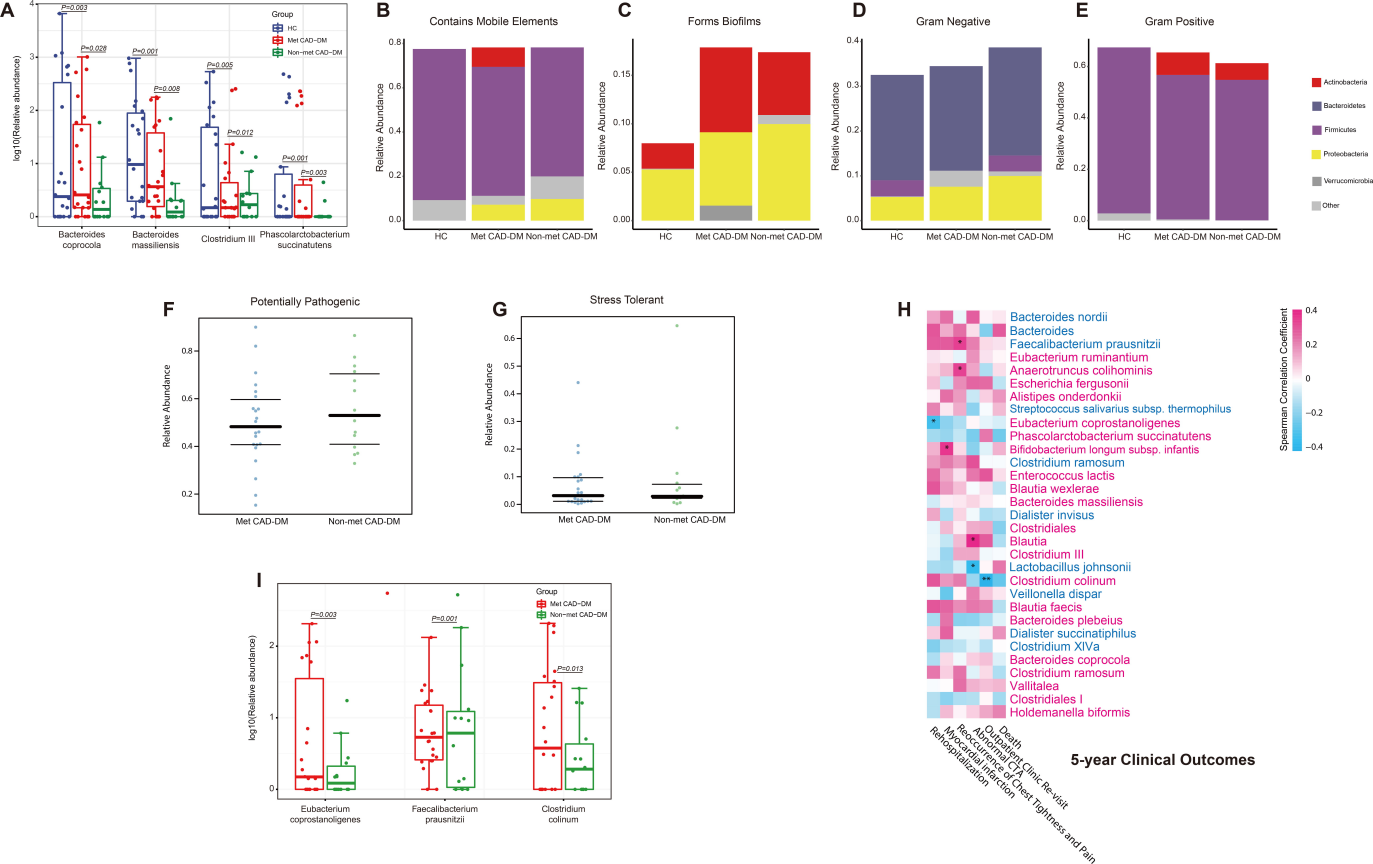
**Metformin changed the abundance of specific microbes and altered the potential function of the gut microbiome, associated with 5-year clinical outcomes**. (A) Abundance of four significantly different species among all three groups. (B) Relative abundance of a form of the bacteria containing mobile elements predicted based on BugBase database. (C) Relative abundance of a form of the bacteria forming biofilms predicted based on BugBase database. (D) Relative abundance of a form of the gram-negative bacteria predicted based on BugBase database. (E) Relative abundance of a form of the gram-positive bacteria predicted based on BugBase database. (F) Relative abundance of potentially pathogenic bacteria predicted by BugBase (FDR-adjusted *p* = 0.43160). (G) Relative abundance of stress-tolerant bacteria predicted by BugBase (FDR-adjusted *p* = 0.81). (H) Spearman correlation between differential gut microbes and 5-year clinical outcomes. The microbes or metabolites are highlighted in red (enriched in Met CAD-DM) and blue (depleted in Met CAD-DM). *FDR-adjusted *p* < 0.05, **FDR-adjusted *p* < 0.01, analyzed by edgeR. (I) The relative abundance of representative harmful and beneficial species. The differential microbes were filtered with FDR-adjusted *p* < 0.05. CTA, computed tomography angiography; FDR, false discovery rate.

The above results showed that the use of metformin modifies the gut microbiome. It is known that the composition and function of gut microbiome are inextricably linked. We carefully analyzed the differences in bacterial composition between the non-Met CAD-DM group and the Met CAD-DM group. We conducted a pairwise comparison of the bacterial composition between the groups. The results indicated that four species of bacteria were significantly different among all three groups. These four bacteria are *Bacteroides coprocola (B. coprocola)*, *Bacteroides massiliensis (B. massiliensis)*, *Clostridium III*, and *Phascolarctobacterium succinatutens (P. succinatutens)* (Fig. 2A). We found that all four bacterial species showed a decreasing trend in the following order: HC group, Met CAD-DM group, and non-Met CAD-DM group.

We further predicted the function of flora using BugBase. The results showed that non-Met CAD-DM group had a higher proportion of potentially pathogenic gut microbiome than the Met CAD-DM group (Fig. 2B). This suggested that the use of metformin may reduce potentially pathogenic bacteria in the intestinal flora. We further analyzed the specific contributions of the gut microbiome to each phenotype. The majority of mobile genetic elements were found to be associated with *Firmicutes*, and their abundance was similar across all three groups (Fig. 2B). The main contributors to the biofilm formation are *Actinobacteria* and *Proteobacteria* (Fig. 2C). The data showed that the two diseased groups have significantly higher levels of biofilms than the HC group. The predominant gram-negative bacteria identified were *Bacteroidetes* and *Proteobacteria* (Fig. 2D) which were most abundant in the non-Met CAD-DM group. In contrast, the predominant gram-positive bacteria were *Actinobacteria* and *Firmicutes* (Fig. 2E) and were the most abundant in the HC group. The results indicated that the non-Met CAD-DM group had a higher proportion of potentially pathogenic gut microbiome than the Met CAD-DM group (Fig. 2F). This suggests that metformin use may reduce the abundance of potentially pathogenic bacteria in the intestinal flora. Furthermore, the findings revealed that the gut microbiota in the Met CAD-DM group exhibited a tendency toward increased, but not significant greater stress tolerance, than the non-Met CAD-DM group (Fig. 2G). This implies that the use of metformin has a potential role in enhancing the tendency of stress resistance of gut flora.

Correlation analysis between gut microbiome composition and 5-year clinical outcomes identified several bacterial species associated with adverse cardiovascular events (Fig. 2H, **Supplementary Table 4**). *Bifidobacterium infantis* was linked to myocardial infarction, while *Faecalibacterium prausnitzii* and *Anaerotruncus colihominis* were associated with recurrence of chest pain. Conversely, *Eubacterium coprostanoligenes*, and *Clostridium colinum* showed protective effects. In addition, we compared the relative abundance of both harmful and beneficial species. We found that metformin use was associated with a significant increase of abundance of beneficial bacteria and significant decrease of harmful species (Fig. 2I).

### 3.4 Serum Metabolic Alterations are Observed in Patients Taking Metformin and This Serum Change is Associated With 5-year Clinical Prognosis

**Fig. 3.**
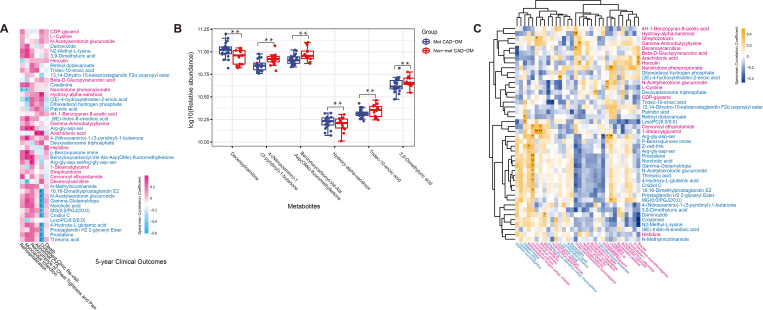
**Spearman correlations between differential serum metabolites and microbes associated with 5-year clinical outcomes**. (A) Spearman correlation between differential serum metabolites and 5-year clinical outcomes. (B) The relative abundance of six key serum metabolites associated with patients’ clinical prognosis. (C) Spearman correlation between differential serum metabolites and differential gut microbes. The microbes or metabolites are highlighted in red (enriched in Met CAD-DM) and blue (depleted in Met CAD-DM). *FDR-adjusted *p* < 0.05, **FDR-adjusted *p* < 0.01, analyzed by edgeR.

In this analysis, we found that significant changes were observed in the gut flora of patients taking metformin. A significant correlation existed between gut microflora alterations and patients’ clinical prognosis. Since alterations in gut flora are known to significantly affect serum metabolomics [34], we further performed an analysis of serum metabolomics. A total of 64 serum metabolites were identified to be associated with moderate alcohol consumption with a threshold of VIP >1 and Wilcoxon rank-sum FDR-adjusted *p* < 0.05 (**Supplementary Table 5**). The score scatter plots of the four modes are shown in **Supplementary Fig. 2**. The metabolic features of three groups (HC, Met CAD-DM, and non-Met CAD-DM) showed differences in score scatter plots. The two disease groups (Met CAD-DM and non-Met CAD-DM) largely overlap across most plots. However, in the lipid negative (LPN) and polar positive (PLP) plot, there is notable distinction between the HC group and the two disease groups. Forty of the differential metabolites can be annotated from the human metabolites database (HMDB). These differential metabolites significantly correlated with clinical outcomes. Spearman correlation analysis showed that some metabolites are positively correlated with clinical outcomes while others are negatively correlated with clinical outcomes (Fig. 3A, **Supplementary Table 6**). Thus, these differential metabolites affected the patient’s prognosis. Six metabolites significantly correlated with clinical prognosis and gut microbiome. These six specific metabolites are: ① Hydroxy-alpha-sanshool (HAS), ② Decenoylcarnitine, ③ 4-(nitrosoamino)-1-(3-pyridinyl)-1-butanone, ④ Tridec-10-enoic acid, ⑤ 3,9-dimethyluric acid, ⑥ Z-vad-fmk (benzyloxycarbonyl-Val-Ala-Asp (OMe)-fluoromethylketone). HAS and decenoylcarnitine both showed higher abundance in the Met CAD-DM group. 4-(nitrosoamino)-1-(3-pyridinyl)-1-butanone, Tridec-10-enoic acid, 3,9-dimethyluric acid, and benzyloxycarbonyl-Val-Ala-Asp (OMe)-fluoromethylketone all showed lower abundance in Met CAD-DM group (Fig. 3B). Furthermore, the change in serum metabolome was associated with alterations in pathways (**Supplementary Fig. 3**). The pathway alterations associated with serum metabolites overlap with those linked to differential gut microbes.

We performed a Spearman correlation analysis to study the association between differential metabolites and differential gut microbiome (Fig. 3C, Table 2, **Supplementary Table 7**). HAS is an unsaturated fatty acid amide [35] that has been reported to attenuate neuronal oxidative stress. HAS can also enhance the antioxidant enzyme activities and inhibit the κB (p65 NF-κB) signaling pathway, which produces inflammatory factors [36]. Furthermore, HAS can also regulate intestinal barrier dysfunction and gut microbiota dysbiosis [36, 37]. It has been reported that decanoylcarnitine is associated with insulin resistance [38] and could inhibit Mmp9 expression [39]. 4-(nitrosoamino)-1-(3-pyridinyl)-1-butanone belongs to the class of organic compounds known as aryl alkyl ketones. Dimethyluric acid has been reported to have pro-inflammatory potential [40]. It has been found that Z-vad-fmk induces non-apoptotic cell death of macrophages which is not beneficial for atherosclerotic plaque stability [41].

**Table 2.** **Lists of key metabolites and its possible functions**.

Metabolite	Possible function	Abundance	Microbiota correlation
Hydroxy-alpha-sanshool	Attenuate neuronal oxidative stress, enhance the antioxidant enzyme activities and inhibit κB (p65 NF-κB) signaling pathway, regulate intestinal barrier dysfunction and gut microbiota dysbiosis	Enriched in Met CAD-DM	Positively associated with *P. succinatutens*
Decenoylcarnitine	Inhibit Mmp9 expression, associated with insulin resistance	Enriched in Met CAD-DM	Positively associated with *P. succinatutens*
4-(nitrosoamino)-1-(3-pyridinyl)-1-butanone	-	Enriched in Met CAD-DM	Negatively correlated with *Eubacterium Coprostanoligenes*
Tridec-10-enoic acid	Long chain fatty acid	Depleted in Met CAD-DM	-
3,9-dimethyluric acid	Have pro-inflammatory potential	Depleted in Met CAD-DM	Negatively correlated with *P. succinatutens*
Z-vad-fmk	Induce non-apoptotic cell death of macrophages and is not beneficial for atherosclerotic plaque stability	Depleted in Met CAD-DM	Negatively correlated with *P. succinatutens*

*P. succinatutens*, *Phascolarctobacterium succinatutens*; Z-vad-fmk, benzyloxycarbonyl–Val–Ala–Asp(OMe)–fluoromethylketone.

In summary, within the Met CAD-DM group, the medication of metformin correlates with the growth of beneficial microbiomes and the decrease of harmful ones. Additionally, the increase in beneficial microbiomes correlates with the reduction of harmful metabolites and an increase in beneficial metabolites. These changes in the serum metabolome further correlates with better cardiovascular health. These correlations may imply a potential mechanism by which metformin improves cardiovascular health and is associated with 5-year clinical prognosis.

## 4. Discussion

Coronary artery disease is closely related to chronic and systemic inflammation [42]. Metformin, a well-known drug used in treating diabetes, has been reported to benefit cardiovascular health [43, 44]. The underlying mechanisms may include enhancing insulin sensitivity and reducing cardiovascular risk factors [45], decreasing inflammation [46], and reducing oxidative stress [47]. Although much research has been performed on metformin and host health, the long-term benefits of metformin on cardiovascular health via gut microbiome have not been studied, especially the long-term benefits associating with the gut microbiome. There have been studies which showed that there was no significant difference in the prognosis between strategies of insulin sensitization and insulin provision. Frye *et al*. [48] compare insulin-sensitizing strategies (including metformin) with insulin-providing strategies in CAD patients and find no significant difference in outcomes, highlighting the need for individualized treatment strategies in this population. Jung *et al*. [49] specifically examined the effects of glucose-lowering agents on CAD outcomes and concluded that metformin significantly reduces the risk of repeat revascularization after PCI in type 2 diabetes mellitus (T2DM) patients. This supports the role of metformin in CAD management but does not address gut microbiota or metabolomics. A meta-analysis by Griffin *et al*. [50] evaluated the cardiovascular outcomes of metformin and concluded that while it is considered safe and potentially beneficial for CAD, evidence from long-term studies remains inconclusive due to limitations in study design and small sample sizes. Our study provides new evidence that long-term metformin use is associated with improved five-year prognosis in CAD patients who have undergone PCI. Despite similar baseline clinical characteristics, patients in the Met CAD-DM group experienced significantly fewer MACE within five years after PCI compared to those without metformin treatment. All the patients included in our study had follow-up for over 5 years. These findings underscore the potential of metformin as a therapeutic strategy for reducing cardiovascular risk in coronary artery disease.

Studies have shown that gut microbiome plays an important role in cardiovascular health and may affect clinical outcomes [51]. It has also been reported that metformin may play important role in alterations of the gut microbiome [52]. Thus, we looked further into the gut microbiome taxonomic features and serum metabolome profiles to reveal the possible associations between metformin intake and better 5-year prognosis. Our study showed that the long-term use of metformin plays an important role in altering gut microbiome taxonomic features. The alpha diversity and beta diversity both presented different taxonomic features between the non-Met CAD-DM group and the Met CAD-DM group.

The alterations of the gut microbiome associated with metformin tends to be beneficial for the patient’s cardiovascular health. It’s worth mentioning that the healthy control group served as a base line reference rather than a focus for comparison. Specific beneficial gut microbiomes (such as *B. coprocola*, *B. massiliensis*, *Clostridium III*, and *P. succinatutens*) had the lowest abundance in the non-Met CAD-DM group and the highest abundance in HC. The Met CAD-DM showed an intermediate abundance of the above four beneficial microbes, which we attributed to the influence of metformin. This suggests that metformin may slow the decrease of these specific beneficial gut microbiota during disease progression. We specially focused on the four gut bacteria: *Bacteroides coprocola (B. coprocola)*, *Bacteroides massiliensis (B. massiliensis)*, *Clostridium III*, and *Phascolarctobacterium succinatutens (P. succinatutens)*. These bacteria exhibited the highest abundance in the healthy controls, followed by the Met CAD-DM group and the non-Met CAD-DM group. This showed a potential association between metformin intake and the abundance of beneficial microbiota. It can be inferred that metformin may be associated with the decrease of these probiotics during disease progression. These four gut florae were reported to be beneficial to host health in several studies. *B. coprocola* was reported to be very promising as a potential preventive and therapeutic agent against obesity [53], which is a risk factor in both CAD and DM [54, 55]. Furthermore, *B. coprocola *was reported to be negatively associated with liver fibrosis among male patients with metabolic dysfunction-associated fatty liver disease (MAFLD) [56]. *B. coprocola* has been demonstrated to have anti-oxidative properties and an improvement in intestinal barrier function [57]. Studies have found that oxidative stress is associated with the onset and progression of coronary heart disease [58, 59] and diabetes mellitus [60, 61]. The imbalance in the generation and clearance of reactive oxygen species (ROS) can lead to extensive and permanent damage, resulting in endothelial dysfunction, and accelerating the occurrence and development of both CAD and DM [61]. These studies indicate that the abundance of *B. coprocola* may correlate with an anti-oxidative effect. Study on *Clostridium III* has shown that it is linked to the attenuation of the NF-κB signaling pathway and the decrease of inflammatory cytokines [62]. Thus, a rise in *Clostridium III* within the Met CAD-DM group may play roles in suppressing the production of inflammation, leading to a better clinical prognosis. Furthermore, *B. massiliensis* and *P. succinatutens*, are capable of producing SCFAs [63, 64], which are positively associated with the use of metformin and contribute to improved disease outcomes. Extensive research has demonstrated that SCFAs possess anti-inflammatory properties [65] and inhibit interleukin (IL)-6 and IL-8 production through G-protein-coupled receptor (GPR)41/43 activation, thereby, reducing systemic inflammation and improving atherosclerosis [66]. Consequently, a higher abundance of *B. massiliensis *and *P. succinatutens *can result in increased production of these protective SCFAs, resulting in benefits for cardiovascular health. Moreover, research has shown that a deficiency of SCFAs is associated with DM [67]. Further research showed that SCFAs are associated with lower blood glucose and lipid levels [68]. Symptoms of diabetes can be alleviated by increasing SCFA-producing bacteria, promoting the production of SCFAs, and upregulating SCFA-glucagon-like peptide (GLP)1/peptide tyrosinetyrosine (PYY)-associated sensory mediators [69]. In recent years, SCFAs such as propionic and butyric acids assembled into nanoparticles and fed into a mouse model of type 2 DM showed improved symptoms [70]. *P. succinatutens*is a predominant gut bacteria, enhancing intestinal epithelial barrier function [71]. It was reported that *P. succinatutens* significantly affected several metabolic pathways such as steroid hormone biosynthesis, primary bile acid biosynthesis, phenylalanine, tyrosine and tryptophan biosynthesis, and phenylalanine metabolism [71]. *P. succinatutens* may play an important role in maintaining a stable symbiosis between the host and core gut microbes. The oral gavage of *P. succinatutens *in mice can improve host organ indexes (including the heart, spleen, and thymus). Additionally, it was reported that *P. succinatutens* significantly increased the crypt depths in the duodenum and ileum, regulating gut mucosal morphology [71]. Thus, metformin may be associated with the alterations of the intestinal microbiome, effectively slowing the depletion of beneficial bacterial species, which may positively influence the prognosis of CAD in patients with DM. Notably the preservation of the four beneficial bacterial species—(*B. coprocola*, *B. massiliensis*, *Clostridium III*, and *P. succinatutens*)—is closely associated with improved metabolic function and reduced disease progression. By mitigating the decrease of these beneficial bacteria, metformin contributes to better disease outcomes.

The bacterial species *Eubacterium Coprostanoligenes (E. coprostanoligenes)* deserves special attention. Metformin is associated with the abundance of *E. coprostanoligenes*, which is linked to a better prognosis of CAD-DM, and metformin is significantly associated with elevated abundance of these beneficial microbes. *E. coprostanoligenes* was reported to be able to alleviate intestinal mucositis by enhancing the intestinal mucus barrier [72]. This enhancement of the intestinal mucus barrier is associated with activating the aryl hydrocarbon receptor/AU-rich element RNA-binding factor 1 (AhR/AUF1) pathway, consequently enhancing Muc2 mRNA stability [72]. The intestinal mucus barrier forms the first line of defense against bacterial invasion while providing nutrients to support microbial symbiosis [73]. On the one hand, the intestinal mucus barrier plays a key role in preventing preclinical diabetes from progressing into diabetes. It was reported that the integrity of the intestinal mucus barrier avoids dysregulated crosstalk between gut microbiota and immune cells, thus preventing the progression of preclinical diabetes [74]. Loss of gut barrier integrity triggers activation of islet-reactive T cells and autoimmune diabetes [75]. On the other hand, the dysfunction of the intestinal mucus barrier is observed in coronary artery disease, and restoration of the intestinal barrier is regarded as a potential therapeutic target in CAD [76]. The improvement of the intestinal barrier’s function also attenuates atherosclerosis by decreasing toxic lipid accumulation and reducing inflammatory cytokines [77]. The dysfunction of the intestinal mucus barrier is also associated with hyperlipidemia [78], which is a well-known risk factor in CAD. Thus, the enrichment of *E. coprostanoligenes *may significantly promote the restoration of the intestinal mucus barrier, thus alleviating autoimmune reactions and improving the prognosis of CAD-DM. This further demonstrates that metformin exerts a protective effect and improves the prognosis of patients suffering from coronary artery disease complicated with diabetes mellitus.

Not only does the gut microbiome affect the 5-year prognosis of CAD-DM patients, but the gut microbiota-derived metabolites also play an important role in the prognosis of the disease. HAS is positively related to *P. succinatutens*, which is regarded as a beneficial microbe. Furthermore, HAS is negatively correlated with rehospitalization and death. Thus, HAS is potentially beneficial to CAD-DM patients and improves the 5-year prognosis of CAD-DM. It has been reported that HAS regulates gut microbiota and metabolites by affecting lipid and amino acid metabolism pathways [37, 79]. Lipid metabolism plays an important role in CAD. Regulating lipid metabolism may influence the prognosis of CAD. Study has shown that the intervention of HAS could also improve the intestinal and metabolic functions [37]. HAS has also been reported to exert anti-diabetic effects by increasing glycogen synthesis through regulation of phosphoinositide-3-kinase/protein kinase B/glycogen synthase kinase-3β/glycogen synthase (PI3K/Akt/GSK-3β/GS) signaling [80]. Additionally, HAS exhibitsvarious pharmacological properties, including analgesia effects and regulating gastrointestinal function [81]. Therefore, HAS plays an important role in improving the 5-year prognosis of CAD-DM.

Our study showed that decenoylcarnitine, a subtype of acylcarnitine, is significantly negatively related to the incidence of 5-year myocardial infarction. Decenoylcarnitine is also negatively associated with rehospitalization, recurrence of chest tightness and pain, as well as an abnormal computed tomography angiography (CTA) within five years. This implies that the higher the abundance of decenoylcarnitine, the better the 5-year prognosis of CAD-DM. Acylcarnitine is essential in fatty-acid metabolism [82]. Although research has shown that acylcarnitine is associated with the development of heart failure [83] and accelerates the progress of atherosclerosis [84], decenoylcarnitine is differs from acylcarnitine and only a few studies have been done on decenoylcarnitine.

In our study, we also discovered several pathogenic metabolites, including 4-(nitrosoamino)-1-(3-pyridinyl)-1-butanone, Tridec-10-enoic acid, 3,9-dimethyluric acid, and Z-vad-fmk. These metabolites are positively associated with a poorer 5-year prognosis. The enrichment of these four metabolites worsens the 5-year outlook of the disease. Tridec-10-enoic acid is a type of long-chain fatty acid (LCFA). These fatty acids can be derived from food or produced by certain types of gut microbiomes such as *Fusimonas intestini*, a commensal species of the family *Lachnospiraceae* [85]. Thus, LCFAs are closely associated with the gut microbiome. LCFAs are also recognized as significant triggering factors for inflammatory disease because they regulate the palmitoylation of signal transducer and activator of transcription 3 (STAT3) through cluster of differentiation 36 (CD36)-mediated endocytosis [86]. Both CAD and DM are closely associated with chronic inflammation. Consequently, LCFAs play an important role in promoting inflammation in CAD and DM, thereby worsening the 5-year prognosis. Furthermore, LCFAs also contribute to M1 macrophage polarization by activating NLRP3 inflammasome and the NF-κB pathway [87]. The accumulation of macrophages and inflammation in atherosclerotic plaques exacerbate atherosclerosis [88], which is the underlying pathological mechanism of CAD. Macrophage polarization is closely associated with hepatic injury in diabetes by activating the protein tyrosine phosphatase 1B/signal transducer and activator of transcription 6 (PTP1B/STAT6) axis [89]. Thus, the polarization of macrophages by LCFAs aggravates both CAD and DM. Our results align with findings from other studies. Tridec-10-enoic acid, which belongs to the family of LCFAs, may be potentially pathogenic to CAD-DM patients and worsen the 5-year prognosis by promoting inflammation and cytokine accumulation. Tridec-10-enoic acid may also be considered a potential drug target.

We focused on two specific metabolites: 3,9-dimethyluric acid and Z-vad-fmk. Both these metabolites exhibited a significant negative correlation with *P. succinatutens*, which is a well-known beneficial microbe. 3,9-dimethyluric acid is a type of dimethyluric acid that has been linked to lipid metabolism and is known to have pro-inflammatory properties [40]. Increased exposure to inflammatory cytokines can activate the ROS-p38-p65 signaling pathway. This activation leads to endothelial cell dysfunction, resulting in coronary atherosclerotic lesions and plaque rupture [90]. Consequently, higher levels of dimethyluric acid contribute to systemic inflammation, worsening the prognosis of patients with CAD-DM. In contrast, Z-vad-fmk, has been shown to induce autophagy and necrotic cell death in smooth muscle cells via macrophage activity. Research indicates that Z-vad-fmk-treated macrophages overexpress and secrete various chemokines and cytokines, including TNF-α. The combination of z-vad-fmk and TNF-α results in smooth muscle cell necrosis. Therefore, Z-vad-fmk is detrimental to atherosclerotic plaque stability as it stimulates inflammatory responses and indirectly induces the death of smooth muscle cells, leading to a worse prognosis in CAD-DM [41]. Thus, higher levels of Z-vad-fmk lead to decreased atherosclerotic plaque stability and result in a worse prognosis of CAD-DM Our results suggest that metabolites such as HAS and decenoylcarnitine may improve the 5-year prognosis of CAD-DM while 4-(nitrosoamino)-1-(3-pyridinyl)-1-butanone, tridec-10-enoic acid, 3,9-dimethyluric acid, and Z-vad-fmk may worsen the prognosis of CAD-DM over the same period.

**Fig. 4.**
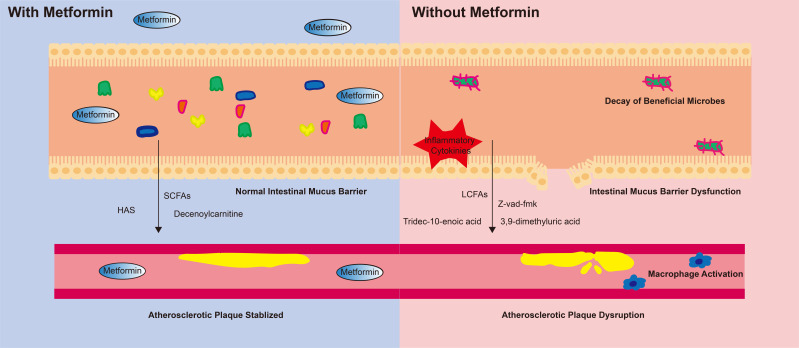
**The potential mechanism of metformin’s beneficial effect on cardiovascular health**. Metformin reshaped the gut microbiome by increasing the abundace of beneficial microbes and preserving the diversity of the gut microbiome. This change further maintained the stability of the intestinal mucus barrier and inhibited the production of inflammatory cytokines. The microbiome-derived metabolites helped stabilize atherosclerotic plaques. In contrast, individuals not taking metformin experienced disrupted gut microbiota diversity. The intestinal mucus barrier exhibited dysfunction, and the production of inflammatory factors was not inhibited. Moreover, the microbiome-derived metabolites promoted macrophage activation and further led to atherosclerotic plaque disruption. SCFAs, short-chain fatty acids; LCFAs, long-chain fatty acids; HAS, hydroxy-alpha-sanshool.

Our results collectively demonstrate that metformin improves the 5-year prognosis of CAD-DM by influencing the gut microbiome. The potential mechanisms outlined in our analysis (Fig. 4) are as follows: (1) Metformin may play important role in influencing the abundance of beneficial microbes such as *B. coprocola*, *B. massiliensis*, *Clostridium III*, and *P. succinatutens*. (2) Metformin promotes the enrichment of SCFA-producing microbes while depleting pathogenic microbes. (3) Changes in the gut microbiome not only directly affect the 5-year clinical prognosis of CAD-DM, but also impact these outcomes by regulating the serum metabolome. (4) After metformin use, the serum metabolome shows higher levels of anti-oxidant metabolites and lower levels of pro-inflammatory metabolites. These, alterations in the gut microbiome and serum metabolome are significantly associated with lower levels of systemic inflammation, diminished macrophage activation, reduced apoptosis of smooth muscle cells, and increased atherosclerotic plaque stability. These cascading effects linked to metformin contribute to a better 5-year prognosis in patients with CAD-DM.

Our study emphasizes the long-term effects of metformin on the gut microbiome. While recent research has highlighted the interaction between metformin and the gut microbiome, it has largely overlooked its long-term impact. Although short-term use of metformin does alter the gut microbiome, these changes are minor and do not significantly affect disease prognosis. Only the long-term use of metformin and its lasting effects are convincing. The prolonged interaction between metformin and the gut microbiome leads to a state of homeostasis that ultimately influences clinical outcomes.

Several limitations of this study need to be acknowledged. First, the sample size of patients was relatively small and the patients were all from a single geographic area. This may affect the interpretation of gut microbiota. Second, the study population included both CAD and DM. Hence, there are two distinct disease variables. The age and BMI of the healthy control groups seemed to be younger from the diseased group. Third, untargeted metabolomics had limited accuracy in the annotation of serum metabolites. The use of Greengenes database also has limited accuracy. We also used FDR <0.2 in the whole calculation. In addition, we did not collect the individual medications (such as ACE inhibitor, beta blocker, etc.) for coronary artery disease. This study also lacked a PICRUSt2 analysis. Experimental research in animals needs to be performed to further establish the relationship between metformin and these alterations in gut microbiota and the serum metabolome.

## 5. Conclusions

In conclusion, CAD patients who have been on long-term metformin therapy demonstrated a significantly improved prognosis within five years following PCI. This protective effect of metformin is further associated with gut microbiome and microbiome-associated metabolites. Multi-omics analysis revealed that metformin is strongly associated with the preservation of beneficial gut microbiota such as *B. coprocola*, *B. massiliensis*, *P. succinatutens*, and *E. coprostanoligenes. *Changes observed in serum metabolome exhibit anti-inflammatory properties, characterized by an increase in HAS and decenoylcarnitine and a decrease in Tridec-10-enoic acid, 3,9-dimethyluric acid, and Z-vad-fmk. These modifications in the gut microbiome and serum metabolome induced by metformin demonstrated beneficial effects on the five-year prognosis for CAD-DM patients on metformin therapy.

## Availability of Data and Materials

The dataset supporting the results of this article has been deposited in the Sequence Read Archive under BioProject accession code SRP167862 (https://www.ncbi.nlm.nih.gov/sra).

## Supplementary Material

Supplementary material associated with this article can be found, in the online version, at https://doi.org/10.31083/RCM26835.
