# Supplementary material for: Long-term Metformin Alters Gut Microbiota and Serum Metabolome in Coronary Artery Disease Patients After Percutaneous Coronary Intervention to Improve 5-year Prognoses: A Multi-omics Analysis
Source: Rev Cardiovasc Med. 2025 May 27;26(5):26835. doi: 10.31083/RCM26835 (PMC12135650; doi:10.31083/RCM26835)
Supplement: Supplementary file 1 [file 2153-8174-26-5-26835-s1.zip › Supplementary Fig. 2.pdf]

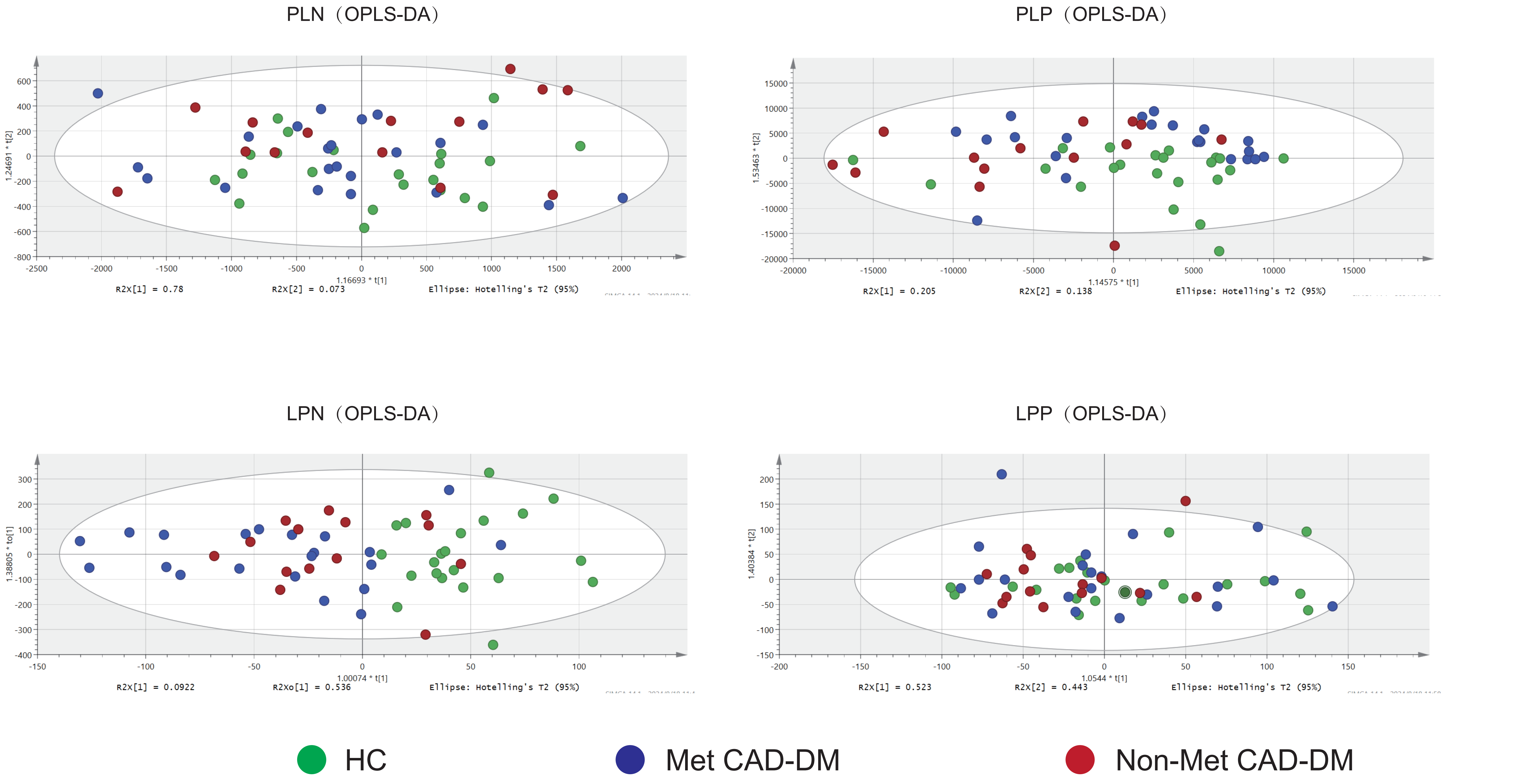

Supplementary Figure S2. Serum metabolites profiles of the three groups. Separation of serum metabolites in polar ionic negative (PLN) mode, polar ionic positive (PLP) mode, lipid negative (LPN) mode, and lipid positive (LPP) mode, revealed by OPLS-DA modeling.
