# Supplementary figures and images for: Long-term Metformin Alters Gut Microbiota and Serum Metabolome in Coronary Artery Disease Patients After Percutaneous Coronary Intervention to Improve 5-year Prognoses: A Multi-omics Analysis
Source: Rev Cardiovasc Med. 2025 May 27;26(5):26835. doi: 10.31083/RCM26835 (PMC12135650; doi:10.31083/RCM26835)

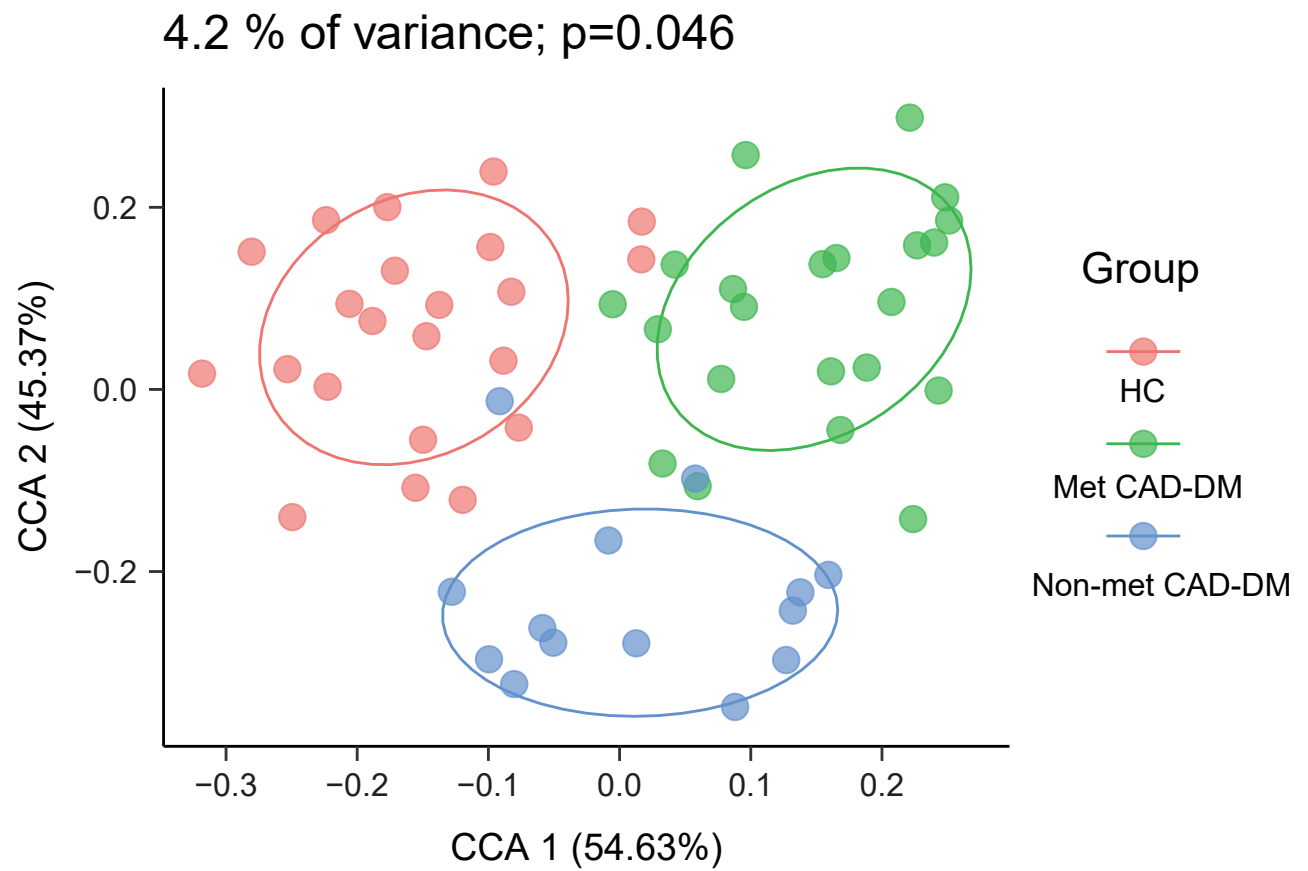

**Figure S1.** Beta diversity was analyzed by CPCoA plot based on Bray-Curtis distances ( $P = 0.046$ , Adonis Test).

Supplement: Supplementary file 1 [file 2153-8174-26-5-26835-s1.zip › Supplementary Fig. 1.pdf]
